# DNA-Metallodrugs Interactions Signaled by Electrochemical Biosensors: An Overview

**DOI:** 10.1155/2007/91078

**Published:** 2007-09-19

**Authors:** Mauro Ravera, Graziana Bagni, Marco Mascini, Domenico Osella

**Affiliations:** ^1^Dipartimento di Scienze dell'Ambiente e della Vita, Università del Piemonte Orientale, Via Bellini 25g, 15100 Alessandria, Italy; ^2^Dipartimento di Chimica, Università di Firenze, Via della Lastruccia 3, 50019 Sesto Fiorentino Florence, Italy

## Abstract

The interaction of drugs with DNA is an important aspect in pharmacology. In recent years, many important technological advances have been made to develop new techniques to monitor biorecognition and biointeraction on solid devices. The interaction between DNA and drugs can cause chemical and conformational modifications and, thus, variation of the electrochemical properties of nucleobases. The propensity of a given compound to interact with DNA is measured as a function of the decrease of guanine oxidation signal on a DNA electrochemical biosensor. Covalent binding at N7 of guanine, electrostatic interactions, and intercalation are the events that this kind of biosensor can detect. In this context, the interaction between a panel of antitumoral Pt-, Ru-, and Ti-based metallodrugs with DNA immobilized on screen-printed electrodes has been studied. The DNA biosensors are used for semiquantitative evaluation of the analogous interaction occurring in the biological environment.

## 1. INTRODUCTION

Chemotherapy is an important weapon for combating cancers. Numerous compounds have been developed as potential candidates for anticancer drugs, but only a handful of them have become effective in clinical protocols. The need of developing new drugs in order to effectively treat various forms of cancer is widely recognized. The development of new drugs requires that the underlying mechanism of action at the cellular and molecular levels has been completely understood.

The potential targets for anticancer drugs are essentially four: nucleic acids, specific enzymes, microtubules, and hormone/growth factor receptors. When nucleic acids are the target, the DNA damage causes cell death (cytotoxic and genotoxic drugs).

There are several mechanisms by which drugs can bind DNA [1], the most well understood being alkylation of nucleophilic sites within the double helix. Most clinically effective alkylating agents have two moieties capable of developing transient carbocations that bind covalently to the electron-rich sites on DNA such as the N7 position of guanine (electrophilic agents). The cross-linking of the two strands of DNA produced by the bifunctional alkylating agents prevents the use of that DNA as a template for further DNA and RNA synthesis leading to inhibition of replication and transcription and, then, to cell death. A large number of chemical compounds are alkylating agents under physiologic conditions, and a variety of such compounds have exhibited antitumor activity. To this category belong “nitrogen mustards” (mechlorethamine, the original “nitrogen mustard,” cyclophosphamide, ifosfamide, melphalan, and chlorambucil), aziridines and epoxides (thiotepa, mitomycin C, and diaziquone), alkyl sulfonates (like busulfan and its analogues), nitrosoureas (carmustine, lomustine, and semustine, above all), triazenes, hydrazines, and related compounds. Moreover, also cisplatin and its congeners are traditionally, albeit improperly, considered alkylating drugs.

A second mechanism of drug binding to nucleic acids is intercalation, that is, the insertion of a planar (generally aromatic) ring molecule between two adjacent nucleotides of DNA. This mechanism is characteristic of many antitumor antibiotics, such as daunorubicin and doxorubicin. The antibiotic molecule is noncovalently, although firmly, bound to DNA and distorts the shape of the double helix resulting in inhibition of DNA replication and RNA transcription.

Finally, a third mechanism of DNA damage is illustrated by bleomycins. These glycopeptides intercalate between guanine-cytosine base pairs of DNA. The end of the peptide binds Fe(II), able to catalyze the reduction of molecular oxygen to superoxide or hydroxyl radicals, that produce DNA strand scission by oxidative stress [2].

Due to the stringent relationship between DNA-drug interaction and *potential* antitumor effect, molecular recognition of nucleic acids by low molecular weight compounds is an area of fundamental interest. A detailed understanding of the interaction of small molecules with DNA is very important in pharmacology.

In this context, cisplatin (*cis* -diamminedichloroplatinum(II), [Pt(NH_3_)_2_Cl_2_]) probably represents a milestone in the antitumor chemotherapy. The activity of cisplatin was serendipitously discovered in 1969 by Rosenberg and colleagues while studying the effects of electric current on bacterial growth. Cisplatin entered clinical trials in the early 1970s and was found to have significant antitumor activity against testicular, ovarian, bladder, and head and neck cancer. Because of the nephro- and neuro-toxicity of cisplatin, there have been intensive efforts to devise analogues with similar or improved pharmacological characteristics. Carboplatin, *cis* -diammine-(1,1-cyclobutanedicarboxylato)platinum(II) (approved worldwide in 1992), shows an antitumor activity similar to that of cisplatin, but with reduced systemic toxicity (better therapeutic index), while oxaliplatin (1R,2R-diaminocyclohexane)oxalatoplatinum(II) (approved for clinical use worldwide in 2003) is effective against colorectal tumors, which are nonsensitive to cisplatin. The consciousness that cisplatin readily reacts with DNA, and that this reaction is crucial in the antitumor activity, focused a great attention in the field of the interaction between metal complexes and biomolecules. Today, forty years after the discovery of the properties of cisplatin, there is no other metal, that is, better understood in its reactivity toward DNA than platinum. Moreover, despite the enormous amount of other metal complexes tested, cancer chemotherapy using metallopharmaceuticals is still largely dominated by platinum compounds [3]. The exploration of nonplatinum metal complexes for use as anticancer agents was initiated in attempts to find less toxic and more specific drugs. In this framework, some ruthenium and titanium complexes have offered the most encouraging results [4]. The imidazolium *trans* -[tetrachlorodimethylsulfoxideimidazoleruthenate(III)] (NAMI-A, [ImH], [*trans*-RuCl_4_(DMSO)Im], Im = imidazole) failed the classical screens of putative anticancer agents, but prevented the development and growth of pulmonary metastases in solid tumors [5–8], and recently has successfully completed Phase I Clinical Trials [9]. The closely related compound, indazolium *trans*-[tetrachlorobis(1*H* -indazole)ruthenate(III)] (KP1019, [IndH], [*trans*-RuCl_4_Ind_2_], Ind = indazole) induces apoptosis in colorectal carcinoma cells and it is currently in Phase I Clinical Trials [10]. Two titanium compounds [11], namely titanocene dichloride [TiCl_2_Cp_2_][12, 13] and budotitane, a *β* -diketonate derivative, [*cis*-diethoxybis(1-phenylbutane-1,3-dionato)titanium (IV)], [14], reached Phase II and Phase I Clinical Trials in Germany, respectively. The general mechanism of action of these nonplatinum compounds is not completely understood yet, but many observations point out that DNA may not be the primary target of these classes of compounds [15, 16].

Characterization of DNA-adducts generally requires a combination of chemical and biological techniques to obtain structural information and to assess the extent and the nature of specific type of binding to DNA, in terms of dissociation constant, stoichiometry, and kinetic constant. Methods able to evaluate the presence of any interaction and, in some cases, to calculate the binding parameter can be classified as mixture- and separation-based methodology. The mexture-based type includes UV absorption and fluorescence [17], nuclear magnetic resonance (NMR, [18]), and Raman spectroscopy [19], mass spectrometry (MS, [20]), calorimetry [21], and surface plasma resonance [22]. The separation-based methods include dialysis, ultrafiltration, ultracentrifugation, chromatography (liquid chromatography and thin-layer chromatography), and electrophoresis (planar and capillary electrophoresis [23, 24]). The last two separation methods are generally combined with sensitive detection techniques (hyphenated techniques), such as MS.

Among the physicochemical techniques, there has been a growing interest in electrochemical investigations. Compared to other methods, electrochemical techniques are characterized by simplicity and reliability and require small amounts of sample, thus offering advantages over biological and chemical assays. Since many small molecules exhibit redox activity, electrochemical method should provide a useful complement to the previously listed methods of investigation.

The electrochemical method is mainly based on the differences in the redox behavior of the nucleic acid-binding molecules in the absence and presence of DNA—including the shifts of the formal potential of the redox couple and the decrease of the peak current resulting from the dramatic decrease in the diffusion coefficient after association with DNA (solution electrochemical methods) (see [25] for a recent review).

On the other hand, since the discovery of the electrochemical activity of nucleic acids by Paleček at the end of the 1950s [26], also DNA has been on the focus of the electrochemical techniques. The binding of drugs to DNA has been described by means of the variation of the oxidation peak current of the electroactive nucleobases, such as guanine and adenine, in the presence of the interacting species.

According to a recent IUPAC document [27], a biosensor is defined as a specific type of chemical sensor comprising a biological recognition element and a physicochemical transducer. The biological element is capable of recognizing the presence, activity, or concentration of a specific analyte in solution. The recognition may be either a binding process (affinity ligand-based biosensor, when the recognition element is, e.g., an antibody, a DNA segment, or a cell receptor) or a biocatalytic reaction (enzyme-based biosensor). The interaction of the recognition element with a target analyte results in a measurable change in a given property. The transducer converts the change in solution property into a quantifiable signal. The mode of transduction may employ several techniques, including electrochemical, optical, and mass or heat measurements. In our case, the electrochemical DNA biosensor consists of a nucleic acid recognition layer, that is, immobilized over an electrochemical transducer [28]. The signal transducer monitors the change that has occurred as a consequence of the binding, converting this into an electronic signal [29]. Observing the pre- and postelectrochemical signals of DNA-drug interaction provides good evidence for the event. The reproducibility of the experiment is strictly related to the history of the electrode surface. In particular, the preparation of the electrode surface influences the final response. For this reason, the use of disposable, low-cost electrode characterized by high reproducibility overcomes the problem, as far as a new, fresh surface is used in each experiment. Various planar technologies are employed for developing solid-state sensors having the above-said characteristics [30]. Screen printing is especially suitable due to its simplicity, low-cost, high reproducibility, and efficiency in large-scale production. This technology enables the deposition of a thick layer of conductive ink on inexpensive substrates and allows precise pattern control.

Although systematic research in this field started recently, several seminal review articles have already been focused on this topic [31–44].

The use of DNA-based biosensors is not limited to the study of interaction between drugs and DNA, but many other applications have been reported. On one hand, DNA- iosensors have been used to test water, food, soil, fish bile [45], and plant samples for the presence of mutagenic pollutants with binding affinities for the structure of DNA [33, 45–54]; on the other hand, DNA-based affinity biosensors have been used to detect specific oligonucleotide sequences in order to find the presence of genes (or mutant genes) associated with particular human diseases [55]. Both aspects are beyond the focus of this paper and will not be discussed further.

However, specific oligonucleotide sequences may be related to the protective cell mechanisms that act against anticancer drugs (drug resistance). One of the main obstacles in the use of metallodrugs in clinical treatment is the development of resistance. In the case of platinum drugs, many mechanisms have been proposed to explain resistance, suggesting that this phenomenon is multifactorial: decrease of intracellular drug accumulation, faster repair of DNA adducts, and increased activity of intracellular pathways of thiol production, in particular glutathione, metallothionein, and thioredoxin, known to be involved in the detoxification of metals. For these reasons, any information on DNA-binding modes, recognition, and repair of DNA damage may be helpful not only to understand the molecular basis of the repair mechanisms, but also to develop new classes of compounds with improved pharmacological properties [56, 57].

## 2. DISCUSSION

### 2.1. Preparation of the screen-printed electrodes (SPEs)

Screen printing is a technique conventionally used in the graphics industry, in the production of circuit boards, or in printing t-shirt designs. When inexpensive, easily mass produced, and, therefore, disposable electrodes for the development of electrochemical biosensors are required, screen-printing is a viable production method. Single use sensors assure avoidance of contamination between samples and constant sensitivity of the different printed sensors. A wide range of different inks (carbon or noble metals-based) and base materials (ceramics or plastic base materials) can be combined to produce electrode systems to suit specific applications.

**Figure 1:**
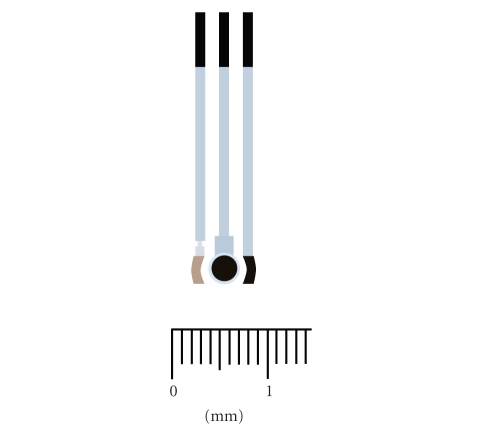
Scan of a screen-printed cell used as electrochemical transducer for the biosensors construction, containing the silver reference electrode (left), the graphite working (centre), and auxiliary electrodes (right).

The planar SPEs used in our laboratories have a three-electrode configuration (Figure 1). They are printed by using inks consisting in finely divided particles of different materials in a blend of thermoplastic resins (silver ink for the reference electrode, graphite ink for working and counter electrodes, while titanium dioxide ink was used for insulating the electrodes).

The sensors were produced in sheets of 80 electrodes. To facilitate handling, the screen-printed electrochemical cells were stuck on a rigid polycarbonate-based support. Each (disposable) electrode can be easily cut by scissors and fits a standard electrical connector [30, 58, 59].

### 2.2. Preparation of the biosensor

As already mentioned in the Introduction, a biosensor is defined as an analytical device, which is capable of providing quantitative or semiquantitative analytical information using a biological recognition element either integrated within or intimately associated with a physicochemical transducer [27]. In our case, the transducer is the SPE while the oxidation peak of guanines is used as the transduction signal for recognize DNA interactions.

The preparation and the following measurement of the interaction at the DNA-modified SPEs include four steps [50]

1. *Electrode activation*: a (mild) surface conditioning step is necessary to oxidize the graphite impurities and to obtain a more hydrophilic surface to favor DNA immobilization (+1.6 V versus Ag-pseudoreference for 120 seconds and +1.8 V for 60 seconds in 0.25 M acetate buffer, containing 10 mM KCl, pH 4.75, under stirred conditions).
2. *DNA immobilization*: in this step DNA is electrochemically accumulated and adsorbed onto the electrode surface by applying a positive potential able to attract negative charged groups of DNA (activated SPE is dipped in a solution of 50ppm calf thymus ds-DNA in 0.25 M acetate buffer with 10 mM KCl, applying a potential of +0.5 V versus Ag-SPE for 5 minutes, under stirred conditions).
3. *Blank or sample interaction*: in this step, the response of the guanine before (reference signal) or after interaction is evaluated (the DNA-modified SPE is dipped for 2 minutes in a solution containing the interacting molecule dissolved in a suitable buffer/saline solution or in the same buffer saline solution without any analyte, to obtain the reference).
4. *Measurement*: a square wave voltammetric (SWV) scan is carried out to evaluate the oxidation of guanine residues on the electrode surface (the height of the guanine peak at +0.95 V versus Ag-SPE was measured in 0.25 M acetate buffer, containing 10 mM KCl).

The four-step protocol is the result of a series of experiments aimed to optimize the final response, in terms of peak height and reproducibility of the signal [50, 60]. The results showed an increase of the sensitivity increasing DNA concentration (until a saturation phenomenon occurred) and the immobilization time (similar results were obtained by other authors. See [61]). The 50 ppm ds-DNA concentration and an immobilization time of 5 minutes (step 2) were chosen as the best compromise for further experiments.

DNA biosensor performances were strongly influenced by the physical properties of DNA (i.e., purity, average chain length, presence of ss-DNA) [48]. Moreover, the solution where the final measurement is performed influences the signal aspect: the acetate buffer gives the best results [48, 50], and this choice was reported also by other authors [62].

**Figure 2:**
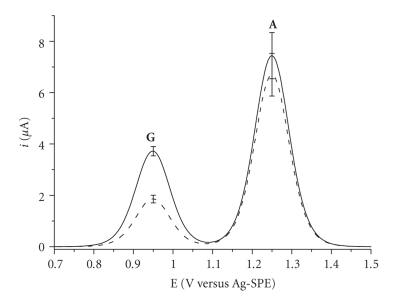
Redox behavior of guanine (**G**, +0.95 V versus Ag-SPE) and adenine (**A**, +1.25 V versus Ag-SPE) bases after an SWV scan carried out with graphite SPE (a baseline correction on the original signals was performed). Note that the signal of DNA alone (solid line) and the decrease of the DNA peaks after the interaction with a general compound able to interact with DNA (dashed line).

We usually estimate the DNA modification due to the interaction with the analyte with the value of the percentage of signal decrease (*S*%). This value is the ratio of the guanine peak height after the interaction of the DNA adsorbed onto the SPE with the analyte (*S*_sample_) and the guanine peak height of the DNA in the buffer solution without drug (*S*_blank_), *S*%=(*S*_sample_/*S*_blank_) × 100. A typical voltammogram is shown in Figure 2.

It must be noted that the two curves of Figure 2 have been obtained from two different SPEs. In fact, only one SWV scan is allowed on each biosensor. If a second SWV is performed, no peak can be observed because of the complete oxidation of the guanine of the immobilized DNA [48]. For this reason, the final *S*% values are expressed as a mean of (at least) three independent measurements. With this procedure, the “memory effect” between one sample and another is avoided. The phenomenon referred to as “electrode fouling,” which is one of the main drawbacks of the electrochemical sensors, is overcome and no calibration is required. Furthermore, the reproducibility of the guanine peak height, calculated over three or more scans on different electrodes is very high, and the standard deviation was estimated to be less than 10%.

It is also possible to study the adenine oxidation peak, but in this case less reproducible signals are obtained (see error bars in Figure 2) [54]. Moreover, this peak is sometimes obscured by the solvent discharge.

Covalent binding with one or both grooves of the double helix, hydrogen and/or van der Waals bonds and intercalation of planar condensed aromatic ring systems between adjacent base pairs (*π* -stacking) are the perturbations that the electrochemical DNA biosensor can detect [63].

### 2.3. Platinum complexes [64, 65]

**Figure 3:**
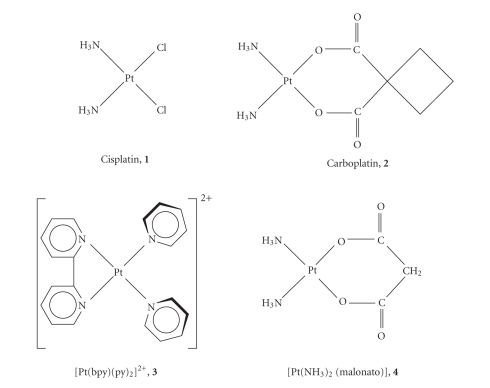
Sketch of the Pt(II) complexes investigated.

Cisplatin, **1** (Figure 3), is administered intravenously for clinical use. In the extracellular environment, the platinum compound experiences high chloride concentration (*∼* 100 mM) and does not undergo appreciable hydrolysis. When cisplatin passes the cell membrane, the reduced intracellular chloride concentration (*∼* 5−10 mM) allows the chloro ligands to be replaced in a stepwise manner by water molecules to form *cis*-[Pt(H_2_O)(NH_3_)_2_Cl]^+^ and *cis*-[Pt(H_2_O)_2_(NH_3_)_2_]^2+^ [66]. It is generally accepted that these two ions are much more reactive than cisplatin and, therefore, react with N-donor ligands, such as DNA nucleobases (the preferred target on DNA is recognized as the guanines having the highest electron density of all four nucleobases), leading to the bending of the DNA structure by 35−40° [67–69]. This key reaction is responsible for the anticancer effect of cisplatin which is able to induce apoptosis/necrosis of the cancer cell [70]. Carboplatin, **2** , undergoes much slower hydrolysis than cisplatin. Since the DNA reactions are primarily limited by the hydrolytic pathways, the reaction between carboplatin and DNA is extremely slow under physiological conditions. For example, the half life of carboplatin reaction with DNA is estimated to be several days [71].

The behavior of these two complexes was compared with that of [Pt(bpy)(py)_2_][PF_6_]_2_, **3** (bpy = bipyridyl, py = pyridine). This complex lacks appropriate leaving groups, so that **3** is devoid of any alkylating properties, but is able to intercalate DNA [72].

**Figure 4:**
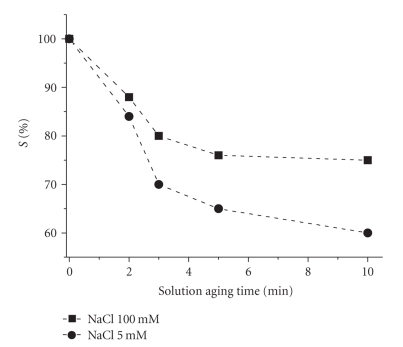
*S*%versus solution aging time for 0.1 mM solution of **1** in unbuffered (pH = 7.4) 5 mM NaCl (circles), and 100 mM NaCl (squares) solutions, respectively.

Figure 4 shows the trend of *S*% values resulting from the interaction between DNA biosensor and a 0.1 mM solution of **1** in 5 mM (intracellular conditions) and 100 mM (extracellular conditions) NaCl, respectively. As expected, the behavior of **1** strictly depends on the concentration of the NaCl and on the aging time of the solution: high concentrations of chlorides inhibit the aquation of cisplatin and, hence, its interaction with DNA.

As far as **1** becomes, after hydrolysis, doubly positive charged species, we have checked whether a simple long-range electrostatic interaction in lieu of an effective coordination to DNA is able to affect the oxidation signal of guanine. For this purpose, we have tested the interaction between the biosensor and solutions containing divalent cations Zn(II) and Cu(II). For both solutions, no variation in the guanine signal was observed (*S*% = 100%).

**Figure 5:**
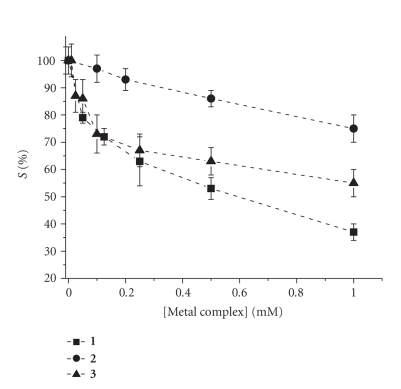
*S*%resulting from increasing concentrations of metal complexes in 0.25 M PB (pH = 7.4) and 5 mM NaCl (interaction time = 2 minutes).

Figure 5 shows the *S*% values resulting from increasing concentrations of metal complexes **1** – **3** in 0.25 M phosphate buffer (PB, pH = 7.4), containing 5 mM NaCl (intracellular conditions).

The interaction increases in a dose-dependent manner, and is stronger for **1** and softer for **2** . Compound **3** shows an initial strong interaction, overimposable to that of **1** , but a minor one at higher concentrations, probably because of the saturation of the intercalating sites on DNA.

**Figure 6:**
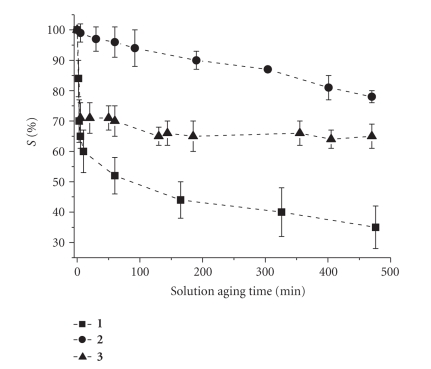
*S*%versus solution aging time for 0.5 mM solution of the metal complexes 1–3 in 0.25 M PB (pH = 7.4) and 5 mM NaCl.

Figure 6 compares the behavior of the three metal complexes in identical experimental conditions (in particular at the same concentration) when the solution aging time is varied. As expected, a stronger effect of solution aging time on *S*% is observed for **1** , while, in the case of **3** , hydrolysis is not required. In fact, this complex does not need to dissociate any ancillary ligand to exert its activity.

Compounds **1** and **2** produce the same electrophilic agent upon hydrolysis, namely [Pt(H_2_O)_2_(NH_3_)_2_ ]^2+^, nevertheless, Figures 5 and 6 reveal that the interaction of carboplatin is much lower than that of cisplatin because of the different rate of hydrolysis (*t*_1/2_ in chloride-free phosphate at 37°C is about 450 hours for **2** [71] compared with 2 hours for cisplatin [73]).

**Figure 7:**
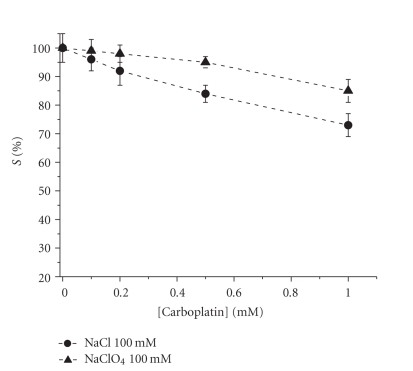
*S*%obtained with different concentrations of carboplatin in 100 mM NaCl or 100 mM NaClO_4_, respectively.

**Figure 8:**
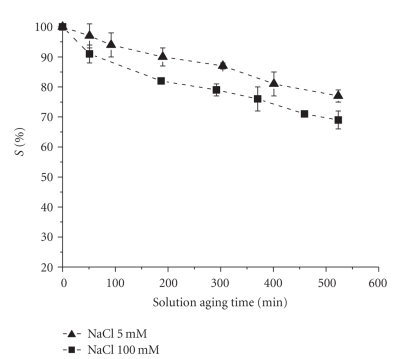
*S*%versus solution aging time for 0.5 mM solution of **4** in 5 or 100 mM NaCl, respectively, (previously unpublished data).

In the case of **2** , the rate of hydrolysis, and hence the interaction with DNA, is increased in the presence of chlorides. In fact, an exchange between the 1,1-cyclobutanedicarboxylato ligand and chlorides in solution is able to transform **2** in **1** [74] that, in turn, undergoes quick activation by aquation. This effect is negligible in the presence of weaker Lewis bases, for instance perchlorates (Figure 7). The exchange reaction is time-dependent as observed in both in **2** and in its malonato-analogue (*cis*-diamminomalonatoplatinum, [Pt(NH_3_)_2_(malonato)], **4**), and increases with [Cl^−^] (Figure 8).

The biosensor may also be used to differentiate the intercalating from the covalent interactions. In fact, by using the same experimental procedures previously described, it is possible to adsorb single-stranded DNA onto the SPE [48]. Similar concentrations of compound **3** gave lower *S*% values (i.e., higher interaction) on the ds-DNA- versus the ss-DNA-based sensor (*S*% = 65 ± 3 versus 92 ± 3, resp.), enforcing the experimental data that identify this complex as an intercalator in lieu of a coordinating agent.

### 2.4. Ruthenium complexes: NAMI-A

**Figure 9:**
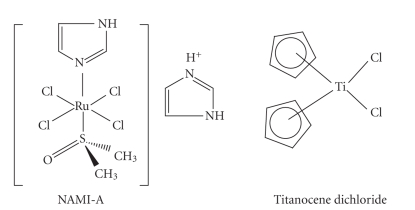
Sketch of the non-Pt(II) complexes investigated.

The complex NAMI-A, (H_2_Im)[*trans*-Ru(III)Cl_4_(DMSO)-(Im)], is a pseudo-octahedral complex with four equatorial chloride ligands and the heterocyclic bases and DMSO as axial ligands (Figure 9).

The complex loses its chloride ligands and transforms into the corresponding, more reactive, aquated species [4] able to bind irreversibly to DNA, albeit this binding is weaker than for similar platinum complexes [75]. In fact, Gallori et al. showed that NAMI-A interacts with DNA at concentrations significantly higher than those at which cisplatin produces similar effects [76]. On the other hand, tight binding of NAMI-A to proteins has been described [64, 77, 78] and it is likely to conceive that the mechanism underlying the antimetastatic activities of NAMI-A does not involve DNA binding as the most significant process, but, perhaps the inhibition of the matrix metallo-proteinases MMP-2 and MMP-9 [8].

**Figure 10:**
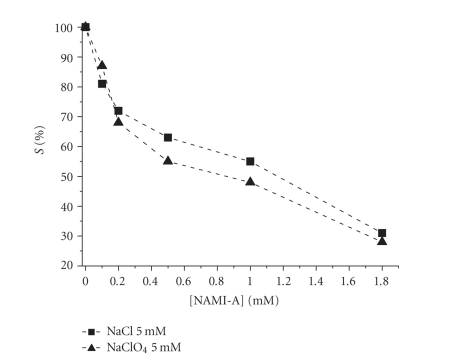
*S*%versus [NAMI-A] in 5 mM NaCl (squares) and 5 mMNaClO_4_ (triangles) solutions, respectively.

Also in the case of NAMI-A, *S*% value decreases as concentration increases (Figure 9), but the concentration of the supporting electrolyte plays minor roles (Figures 9 and 10). In fact, NaCl, that should exert mass effect, and NaClO_4_, that produces the noncoordinating perchlorate anion, gave similar results (Figure 9) [64]. Indeed, it is known in literature that chlorides have a minor effect over NAMI-A aquation [79]. NAMI-A shows higher *S*% values in comparison with **1** , especially at low chloride concentration. These experimental data further reinforce the hypothesis that DNA is not the preferential targets of NAMI-A.

### 2.5. Titanium complexes

**Figure 11:**
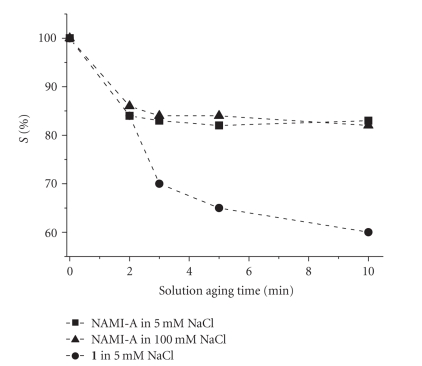
*S*%versus solution aging time for 0.1 mM solution of NAMI-A in 5 mM NaCl (squares) and 100 mM NaCl (triangles) solutions, respectively, compared to the trend of the same concentration of **1** in 5 mM NaCl (circles).

Unlike the very well-studied platinum complexes, interactions of Ti complexes with DNA are poorly understood. It seems that titanocene dichloride TiCp_2_Cl_2_ (Figure 11) is able to interact with transferrin, the protein associated with iron transport. In this form, titanium active species could cross the cell membrane, but the nature of the actual cytotoxic species remains unknown [15].

In literature, there are conflicting results about whether titanocene dichloride binds DNA or not. Some reports have suggested that TiCp_2_Cl_2_ does not bind nucleotides and oligonucleotides at physiological pH [80–82], but there is experimental evidences of titanium being accumulated in the cellular nucleic acid-rich regions, particularly in the chromatin [83]. Recently, it has been shown that TiCp_2_Cl_2_ interacts weakly with nucleotides at neutral pH through the phosphoesters most probably as bare Ti(IV) species [84].

These conflicting results about titanocene dichloride binds DNA prompted us to test if the biosensor were able to give some information about the degree of interaction.

**Figure 12:**
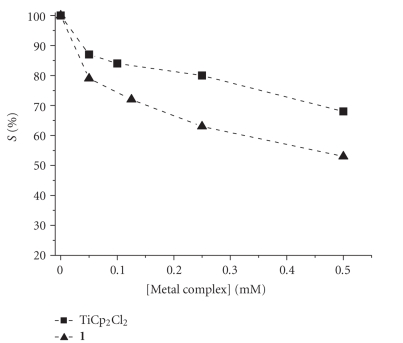
*S*%versus [metal complex] in 0.25 M PB/5 mM NaCl solutions of TiCp_2_Cl_2_ and **1**, respectively.

**Figure 13:**
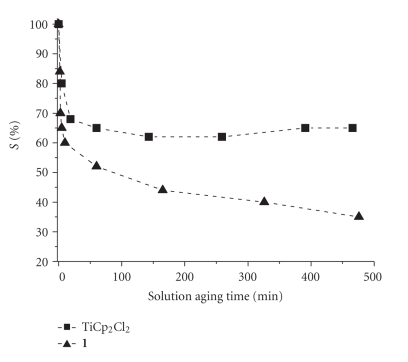
*S*%versus solution aging time for 0.5 mM solution of TiCp_2_Cl_2_ in 0.25 M PB/5 mM NaCl, compared to the trend of the same concentration of **1**.

Figures 12 and 13 show, unequivocally, that TiCp_2_Cl_2_ has a lower degree of interaction with DNA biosensor than cisplatin [65]. The trend of *S*% with solution aging time is almost constant. These two points fit with the literature data showing that the hydrolysis of TiCp_2_Cl_2_ proceeds much faster than cisplatin: the half-life of the first aquation of chloride ligand is too fast to be measured and the second aquation step has a *t*_1/2_ ≈ 50 minutes [80]. Therefore, we expect that both the active species [TiCp_2_(H_2_O)Cl]^+^ and [TiCp_2_(H_2_O)_2_]^2^^+^ are present in solution just at the beginning of the experiment. If we accept that the DNA binding occurs at the phosphate groups level, it is evident that an ionic interaction between Ti cation and external phosphate backbone produces a minor effect on the oxidation of G with respect to the direct coordination of N7.

## 3. CONCLUDING REMARKS

In the field of environmental sciences it has been demonstrated the good relationship between genotoxicity of a sample (measured by specific assays like Toxalert) and the presence of substances with high affinity for DNA (measured by the DNA biosensor) [48, 50, 51], but the use of biosensors in pharmacokinetic studies deserves some caution.

It is generally accepted that a direct relationship between cytotoxicity and DNA-bound Pt exists [85–87], but there are also many factors that hamper the DNA platination. The DNA biosensors do not give an “absolute” measure of the genotoxic power of a potential drug as it uses DNA free of histones, not organized in superior structures, and nuclear and cellular membranes are missing. Furthermore, in a cell-free system, the cellular thiols (glutathione and metallothioneins) able to intercept the platinum complexes are not present and other repair mechanisms are missing.

Moreover, the ratio between metal drug and DNA is far from real pharmacological conditions. In fact, in the case of Pt drugs, it has been measured that cytotoxicity occurs when there are around 2−10 nmoles of Pt/g DNA [88]. Our determinations revealed that about 3 × 10^−9^ g of DNA coated the biosensor [65, 89]. This means that the ratio between metal complex and immobilized DNA is incredibly high and in these conditions a large number of compounds could interact with guanine, even without being active antitumor drugs.

However, this procedure can be very useful for a rapid screening of the samples and may be of interest in studying (i) the possible reaction of the metal complex in solution and hence the formation of DNA-active or inactive species by reaction with water or other molecules acting as ligand (i.e., chlorides), and (ii) the strength of perturbation caused directly or through the DNA chain by such metallodrugs on the electron density of N7-G, that is, the real observable in such a measurements.

For the above reasons, the DNA biosensor could give useful and quick information and could be integrated in a panel of tests in order to quickly evaluate and quantifies the affinity of low-weight molecules with DNA.
